# Supplementary material for: Learning curve and surgical outcome of robotic assisted colorectal surgery with ERAS program
Source: Sci Rep. 2022 Nov 29;12:20566. doi: 10.1038/s41598-022-24665-w (PMC9709162; doi:10.1038/s41598-022-24665-w)
Supplement: Supplementary file 2 — Supplementary Information 2. [file 41598_2022_24665_MOESM2_ESM.zip › RAL Raw data-Ñ[▒K/071/OP N_ñw╝╨░O▒Kñσ.pdf]

W55 / W55

臺中榮民總醫院
手術室護理記錄

[redacted]

基本資料

- 室別： 開刀房-17室，手術科別： CRS，手術類別： 常規，日期： 2021/04/08
- 進入時間： 1615 ； 麻醉開始時間： 1635 ； Time Out時間： 1750 ； 劃刀時間： 1750 ； 手術結束時間： 2250 送出時間： 2320 ；
- 麻醉方式： GE
  診斷： Malignant tumor of sigmoid colon SP SUBTOTAL COLECTOMY
  手術名稱： Robot Assisted Colectomy(L)
  手術局部用藥： nil
  術前預防性抗生素：
  - 抗生素名稱一： Veterin 1GM IVD,由麻醉護理師執行
  - 時間： 1655
  - 上次簽章: 陳慧瑜

  , 追加預防性抗生素：
  - 抗生素名稱一： Veterin 1GM IVD,由麻醉護理師執行
  - 時間： 2055
  - 上次簽章: 彭芳渝
- 手術醫師： 蔣鋒帆，林陳彥，陳琬清，林易萱
- 麻醉醫師： VS 張詒婷
-
  - 刷手護士A ： 周筱涵，時間： 1615，刷手護士B ： 林怡廷，時間： 1615，接替刷手一： 陳慧瑜，時間： 1800，接替刷手二： 林佳慧，時間： 2100
  - 流動護士A ： 陳慧瑜，時間： 1615，接替流動一： 余姿儀，時間： 1800，接替流動二： 洪豐麟，時間： 1850
- 更多接替及流動護士： 接替流動三： 彭芳渝，時間： 2030

皮膚狀況

A-V Shunt： 無
術前： 完整
記錄者： 上次簽章: 陳慧瑜
術後： 完整
記錄者： 上次簽章: 彭芳渝

手術準備

手術臥式 單一手術臥式

-
  - 手術臥式： 截石臥式
  - 使用附件： 溫毯 40 ℃，頭圈，腳架，其它： lateral support x2,海綿
  - 皮膚準備： 10%Aq Betadine，Hibiscrub，2% Chlorhexidine
  - 電燒： 使用電燒: 電極片位置： 右大腿，雙極電燒，記錄者： 上次簽章: 陳慧瑜

止血帶T： 無

植入物

- 植入物名稱/號碼/數量/部位： EndoWrist stapler 45-4.3mm Green*2/colon

, 記錄者： 上次簽章: 洪豐麟

標本送檢

- 細菌培養 無
- 細胞學 無
- 冰凍切片 無
- 病理標本
  組織名稱一： distal cut end
  ,

組織名稱二： IMA root

,

組織名稱三： S-Colon

- 截肢 無

標本交付 標本： 3 袋 ，標本： 0 盒 ，標本： 0 瓶 ，細胞學： 0 管 ，Culture Tube： 0 管 ，被交付醫師： CR 林陳彥，

- 記錄者： 上次簽章: 彭芳渝
- 時間： 2230

傷口術後數料

傷口數料： 傷口數料： 無

- 請於圖上標示部位：

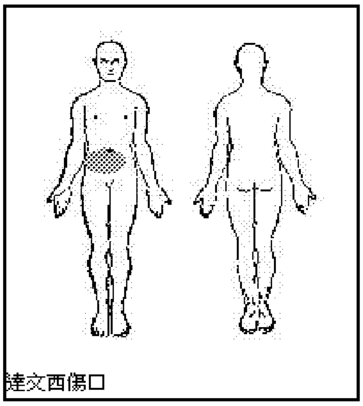

- 傷口數料： meport

導管

導管	天數	導管資訊	插入日期	插入時間	更換日期	拔除日期	拔除時間	拔除原因	預計拔除/更換	預計拔除/更換日期
Epidural PCA(1)	1	部位:硬脊髓;	2021/04/08	1600						
Foley(1)	1	號碼:16 Fr.; 部位:膀胱; 註記:balloon10cc;	2021/04/08	1650						
A-line (1)	1	號碼:20 G; 部位:左手;	2021/04/08	1630						
Double Lumen CVC(1)	1	號碼:8 fr; 深度:15cm; 部位:jugular (右); 象限:RU;	2021/04/08	1645						

用物計數

- 手術中出血量： minimal c.c.
- 手術中尿量： 750 c.c.
-
    - 其它： 無

用物計數： 多切口同時縫合

- 1.器械： 正確，2.縫針： 正確，3.刀片： 正確，4.紗布： 正確，5.腹部墊： 未使用，6.紗球： 未使用，7.PEANUTS ： 未使用，8.紗條： 未使用，9.絲帶： 未使用，10.針頭： 未使用，11.Vessel loop： 未使用，12.Boots： 未使用，13.Jelco： 未使用，14.棉條： 未使用，
    - 第一次：
        - 刷手護士 ： 林佳慧
        - 流動護士： 上次簽章: 彭芳渝
        - 時間： 2200

,

    - 第二次：
        - 刷手護士 ： 林佳慧
        - 流動護士： 上次簽章: 彭芳渝
        - 時間： 2220

,

    - 第三次：

- 刷手護士 ： 林佳慧
- 流動護士： 上次簽章: 彭芳渝
- 時間： 2230

備註

備註：XI 30度scope no1

- 記錄者： 彭芳渝 上次簽章: 陳慧瑜
- 時間： 1615

填寫護理人員 彭芳渝

正式摘要

W55 / W55

doctorRank:B
